# CD44 Expression in Renal Tissue Is Associated with an Increase in Urinary Levels of Complement Components in Chronic Glomerulopathies

**DOI:** 10.3390/ijms24087190

**Published:** 2023-04-13

**Authors:** Natalia Chebotareva, Anatoliy Vinogradov, Larisa Tsoy, Vladimir Varshavskiy, Ekaterina Stoljarevich, Anna Bugrova, Yulia Lerner, Tatyana Krasnova, Evgeniya Biryukova, Alexey S. Kononikhin

**Affiliations:** 1 Department of Nephrology, Sechenov First Moscow State Medical University, Trubezkaya, 8, 119048 Moscow, Russia; 2 Institute for Clinical Morphology and Digital Patology, Sechenov First Moscow State Medical University, Trubezkaya, 8, 119048 Moscow, Russia; 3 Department of Internal Medicine, Lomonosov Moscow State University, GSP-1, Leninskie Gory, 119991 Moscow, Russia; 4 Morphology Department, Evdokimov Moscow State University of Medicine and Dentistry, Delegatskaya Str., 20, 127473 Moscow, Russia; 5 Emanuel Institute for Biochemical Physics, Russian Academy of Science, Kosygina Str., 4, 119334 Moscow, Russia; 6 Skolkovo Institute of Science and Technology, Bolshoy Boulevard 30, Bld. 1, 121205 Moscow, Russia

**Keywords:** CD44, parietal epithelial cells, complement, renal fibrogenesis

## Abstract

It is suggested that activated CD44+ cells play a profibrogenic role in the pathogenesis of active glomerulopathies. Complement activation is also involved in renal fibrogenesis. The aim of the study was to evaluate the role of the activation of CD44+ cells in the kidney tissue and complement components’ filtration to the urine as factors of renal tissue fibrosis in patients with glomerulopathies. In total, 60 patients with active glomerulopathies were included in our study: 29 patients with focal segmental glomerulosclerosis (FSGS), 10 patients with minimal change disease (MCD), 10 patients with membranous nephropathy (MN), and 11 patients with IgA nephropathy. The immunohistochemical peroxidase method was used to study the expression of CD44+ in kidney biopsies. Components of complement were analyzed in urine by the multiple reaction monitoring (MRM) approach using liquid chromatography. Strong CD44 expression was noted predominantly in PEC and mesangial cells (MC) in patients with FSGS, and to a lesser extent, in patients with MN and IgA nephropathy, and it was absent in patients with MCD. Expression of profibrogenic CD44+ in glomeruli correlated with the levels of proteinuria and complement C2, C3, and C9 components, and CFB and CFI in urine. The CD44+ expression scores in the renal interstitium correlated with the level of C3 and C9 components of complement in the urine and the area of tubulo-interstitial fibrosis. The strongest expression of CD44+ was found in the glomeruli (MC, PEC, and podocytes) of patients with FSGS compared with other glomerulopathies. The CD44 expression score in the glomeruli and interstitium is associated with high levels of complement components in the urine and renal fibrosis.

## 1. Introduction

The CD44 proteins form a ubiquitously expressed family of cell surface adhesion molecules involved in cell–cell and cell–matrix interactions.The main physiological role of CD44 is to maintain tissue structure via cell–cell and cell–matrix adhesion. It has been observed that the expression of CD44 isoforms is upregulated in neoplasia and tissue fibrosis [1]. CD44 participates in inflammatory and fibrotic processes such as leukocyte recruitment, and hyaluronic acid (HA) metabolism. CD44 is implicated in fibrillar collagen accumulation and wound healing during the injury response [2]. Prolonged CD44 and CD44–HA interaction can lead to fibrosis and scarring [3]. A considerable body of experimental data is accumulating which show a high profibrogenic potential of CD44-positive cells in kidney diseases. It is associated with the development of interstitial fibrosis and glomerulosclerosis. Their significance in the development of glomerulosclerosis in nephritis with crescents and in primary focal segmental glomerulosclerosis (FSGS) has been established [4,5,6,7]. For example, CD44-positive cells activate parietal epithelial cells (PEC), which is one of the key factors in the development of glomerulosclerosis in patients with chronic glomerulopathies [8,9,10]. CD44 expression promotes parietal epithelial cells (PEC) and mesangial cell (MC) proliferation, facilitates inflammatory cell migration, cell–matrix interactions via hyaluronan binding. Moreover, CD44 also interacts with collagen, laminin, fibronectin, and osteopontin as ligands contributing the accumulation of extracellular matrix [1]. However, the factors which trigger CD44 expression in chronic glomerulopathies in humans remain unknown.

Complement components are suspect factors that have been suggested as triggers for renal tissue fibrosis. In has been shown that urinary C5b-9 is associated with disease activity and unfavorable outcomes in glomerular diseases [11,12]. The detection of increased levels of complement components in plasma and urine has been associated with the development of renal fibrosis and poor prognosis in patients with FSGS [13,14,15]. Our hypothesis is that activated complement factors may be associated with the activation of CD44+ cells and the acquisition of profibrogenic properties by these cells.

The aim of our study was to evaluate the relationship between the urinary excretion of complement components and CD44 expression in the renal tissue as factors associated with renal tissue fibrosis in active chronic glomerulopathies/glomerulonephritis.

## 2. Results

### 2.1. Expression of CD44 by PEC

In the FSGS group, CD44+ PEC expression was observed in 20 (69%) of the 29 kidney biopsies: moderate expression was observed in 10 (34%) patients; mild expression was observed in 10 (34%) patients. In the MCD group, CD44+ PEC mild expression was observed in 3 (30%) of the 10 patients; CD44+ PEC expression was absent in 7 (70%) of the patients. In the MN group, CD44 PEC expression was mild in two (20%) patients; no expression was observed in eight (80%) patients. In the IgA nephropathy group, CD44+ PEC moderate expression was observed in two patients (18%), mild expression was observed in six patients (55%), and three patients (27%) did not have any expression (Table 1).

### 2.2. Expression of CD44 in the Mesangium

In the FSGS group, strong CD44 expression III in the mesangium was observed in 5 patients (17%); it was moderate in 15 patients (52%), mild in 7 patients (24%), and absent in 2 patients (7%). In the MCD group, CD44 moderate expression in the mesangium was observed in two patients (20%); it was mild in seven patients (70%), and absent in one patient (10%). In the MN group, CD44 moderate expression in the mesangium was observed in three patients (30%); it was mild in five patients (50%), absent in two patients (20%). In the IgA nephropathy group, CD44 moderate expression in the mesangium was observed in nine patients (82%); it was mild in one patient (9%), and absent in one patient (9%) (Table 1).

### 2.3. Expression of CD44 in the Interstitial Compartment

In the FSGS group, intensive CD44 expression in the interstitium was observed in 20 patients (69%), and it was moderate in 9 patients (31%). In the MCD group, expression of CD44 in the interstitium III was observed in six patients (60%), and it was moderate in four patients (40%). In the MN group, expression of CD44 in the interstitium III was observed in seven patients (70%), and it was observed in the interstitium II in three patients (30%). In the IgA nephropathy group, CD44 expression III in the interstitium was observed in 10 patients (91%); CD44 expression II was observed in 1 patient (9%).

### 2.4. Relationship between Renal CD44 Expression and the Level of Complement Components in Urine

**Figure 1:**
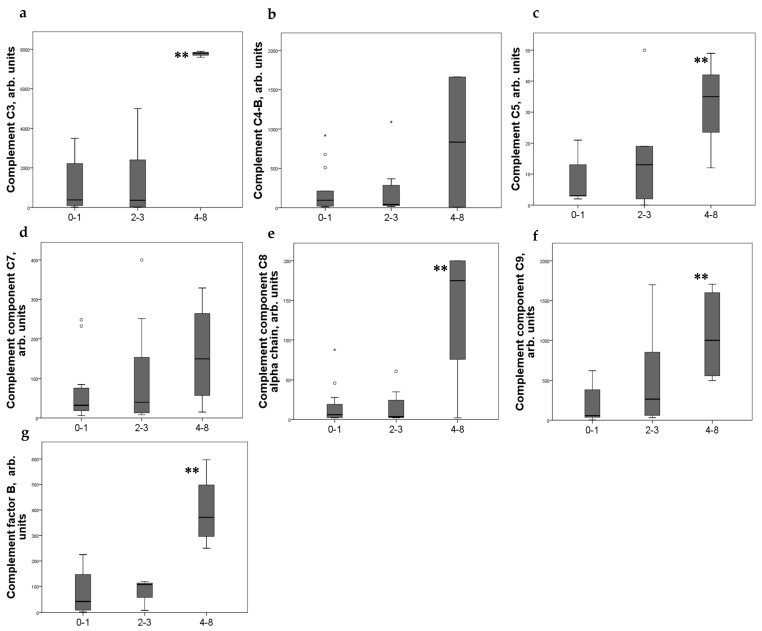
Components of complement in urine (**a**) C3; (**b**) C4-b; (**c**) C5; (**d**) C7; (**e**) C8alfa chain; (**f**) C9; (**g**) factor B of patients with different CD44+ PEC expression score. Boxplots above present the results of Kruskal–Wallis test of parameters for independent samples with different CD44+ expression score. Center line indicates median, top of box indicates the 75th percentile, bottom of box indicates the 25th percentile, whiskers indicate the 10th and 90th percentiles, asterisk indicates extreme values (more than 3 interquartile ranges), and circles indicate outliers (between 1.5 and 3 interquartile ranges). ** *p* < 0.05.

Significant differences in the level of some complement components in urine were noted depending on the CD44 expression scores. Moderate expression of CD44+ in PEC was associated with increased of C3, C5, and C8–9 components of complement and factor B in urine (Figure 1).

**Figure 2:**
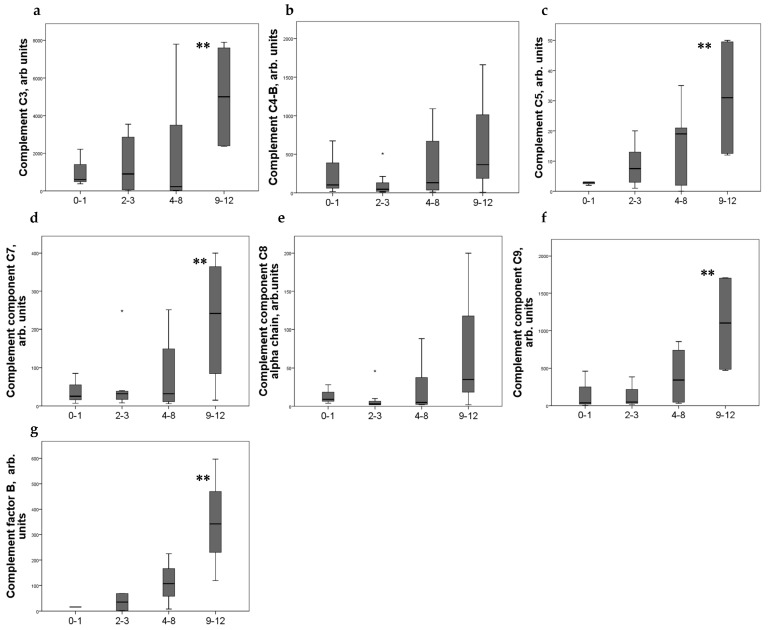
Components of complement in urine (**a**) C3; (**b**) C4-b; (**c**) C5; (**d**) C7; (**e**) C8alfa chain; (**f**) C9; (**g**) factor B of patients with different mesangial CD44 expression score. Boxplots above present the results of Kruskal–Wallis test of parameters for independent samples with different CD44+ expression score. Center line indicates median, top of box indicates the 75th percentile, bottom of box indicates the 25th percentile, whiskers indicate the 10th and 90th percentiles, asterisk indicates extreme values (more than 3 interquartile ranges). ** *p* < 0.05.

**Figure 3:**
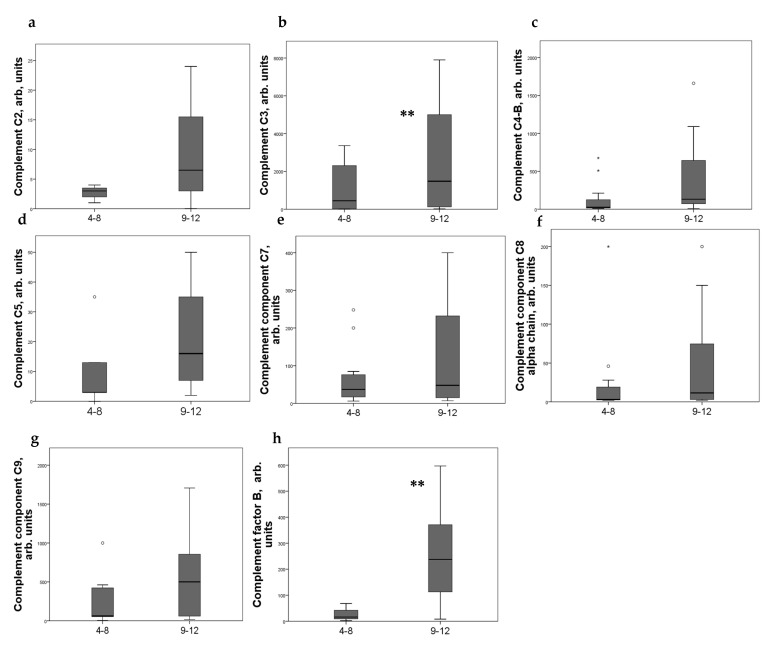
Components of complement in urine of patients (**a**) C2; (**b**) C3; (**c**) C4-b; (**d**) C5; (**e**) C7; (**f**) C8 alfa chain; (**g**) C9; (**h**) factor B with different interstitial CD44 expression score. Boxplots above present the results of Kruskal–Wallis test of parameters for independent samples with different CD44+ expression score. Center line indicates median, top of box indicates the 75th percentile, bottom of box indicates the 25th percentile, whiskers indicate the 10th and 90th percentiles, asterisk indicates extreme values (more than 3 interquartile ranges), and circles indicate outliers (between 1.5 and 3 interquartile ranges). ** *p* < 0.05.

Strong expression of CD44+ in mesangial cells (MC) corresponded to increased excretion of C3, C5, and C9 components of the complement system, as well as factor B. For C4 and C8, a tendency to increase with an increase in CD44 expression was revealed (Figure 2). CD44 expression in the interstitial compartment was higher in patients with increased components of complement in urine (Figure 3).

CD44+ expression in MC was associated with hematuria and % of glomerulosclerosis, and CD44 interstitial expression was associated with the tubule-interstitial fibrosis (TIF) score. Podocytes showed variable expression of CD44, but no significant correlations with these laboratory parameters were found (Table 2).

The creatinine levels/eGFR and the TIF score significantly correlated with the levels of complement components C3, C4b, C5, and C9, but most correlations were found for daily proteinuria levels (Table 3, Supplementary Figure S2).

## 3. Discussion

We studied the expression of CD44+ in the renal tissue of patients with active chronic glomerulonephritis. CD44 is a cell-surface glycoprotein involved in cell–cell interactions, cell adhesion, and migration. Strong and moderate CD44 expression in the PEC and MC we noted mostly in FSGS and IgA nephopathy; we detected mild expression in MCD and MN. Moderate and strong CD44 interstitial expression was found in patients with active nephritis and high proteinuria regardless of histological form.

The CD44 is an antigen that presents on the surface of most cells or tissues, with the exception of platelets, hepatocytes, and cardiomyocytes. It signals to the proteins of the membrane cytoskeleton or nucleus, regulating the expression of various genes associated with adhesion between the cells and matrix, cell migration, proliferation, differentiation, and survival [16]. CD44 regulates extracellular matrix remodeling during wound healing [2], as well as collagen accumulation and scar formation in an ischemic cardiac injury model and an acute lung injury model [17,18]. Therefore, CD44 plays an important role in the processes of inflammation and fibrogenesis.

Strong and moderate CD44 expression has been detected in mesangial cells, podocytes, and PECs, which suggests that resident glomerular cells may acquire a profibrogenic phenotype under the influence of proteinuric and inflammatory components. It has been observed recently that increased CD44 expression leads to a pro-sclerotic PEC phenotype in glomeruli [19]. Data obtained in experimental and human FSGS and diabetic nephropathy showed that PECs typically express CD44, thereby producing PEC-derived extracellular matrix protein isoforms in an activated state [20]. PECs showed the de novo expression of CD44, which allows for the invasion of the glomerular tuft during the scarring of glomerular diseases [5,8]. For example, CD44 is the major receptor of hyaluronan (HA), an important component of the extracellular matrix, and CD44 is markedly enhanced in glomerular crescents [21]. HA displays a number of important proinflammatory effects, including the upregulation of cytokines, chemokines, and adhesion molecules. HA could provide an interstitial matrix along which CD44-positive mononuclear cells could easily migrate, contributing cellular and fibrosis crescents [22]. In an experimental study, PECs of CD44+/+ in mice produce vimentin and α-SMA, that are accompanied by segmental and global glomerulosclerosis [23]. We also detected strong CD44+ expression in PEC in FSGS patients. Apparently, not only PEC play a role in glomerular scarring. Strong expression of CD44 in mesangial cells and podocytes was found in the majority FSGS patients, and in a smaller number of patients with MN and MCD. In patients with active and progressing IgA nephropathy, strong CD44 MC expression was also shown. An experimental study carried out by Nikolic-Paterson DJ et al. supported the expression of CD44 on MC and its interaction with hyaluronan, which mediates cell–matrix interactions and the further accumulation of ECM [24]. In our opinion, not only PEC but also CD44+ MC acquire profibrogenic properties, and they also might be involved in glomerular scarring.

Using mass spectrometry, we found an increase in the level of complement components C3, C4b, C5, C9, and CFB in the urine of patients with active nephritis. Strong correlations between CD44 expression in both the glomeruli and the interstitial compartment and components of complement were found. The correlations between the proteinuria levels, expression score of CD44 in glomeruli, and levels of several complement components indicate that they, being filtered through a damaged glomerular filter, can be activated in the urinary space of Bowman’s capsule and affect resident glomerular and interstitial cells. They seem to be important factors in the activation of inflammation and fibrosis. In a recent publication, we noticed that urinary complement components C9, C4b, and CFB might be used as biomarkers for severe FSGS with steroid-resistant nephrotic syndrome, progressing the disease with the formation of TIF [15].

Based on these results, we suggest the following mechanism for renal tissue fibrosis: complement components with plasma protein leakage into the urinary space; filtered complement can be activated within the urinary space and change the phenotype of glomerular cells (MC, PEC, and podocytes) which begin to express CD44+ and acquire profibrogenic features [25].

Systemic complement activation in patients with high proteinuria can also be involved. Huang J et al., having studied the complement components C3a, C5a, soluble C5b-9, C4d, C1q, MBL, and Bb in the plasma and urine of patients with FSGS, concluded that there was systemic complement activation in these patients. The urinary levels of Bb were elevated, positively correlated with C3a and C5b-9 levels, renal dysfunction, and interstitial fibrosis [13]. These data are confirmed by Thurman JM et al.’s study. Plasma and urine Ba, C4a, and sC5b-9 were significantly higher and correlated with the primary outcome in FSGS patients [14]. Another group of authors also noted that the level of C3 mRNA was also upregulated in the tubulointerstitial tissues of FSGS patients [26,27].

Our study has limitations. The number of patients was relatively small. Validation in more cases is required to consider the identified correlations. The results presented are preliminary.

In conclusion, we suggest that the intensity of fibrogenesis in the glomeruli and renal interstitium can be mediated by activated resident CD44+ renal cells under the local influence of urinary complement components. Patients with persistent activity, high proteinuria or nephrotic syndrome, were exposed to the prolonged contact of glomerular and interstitial cells with complement components and the progression of renal fibrosis.

The role of CD44 as an important factor in the development of renal tissue fibrosis in chronic progressive forms of glomerulopathies requires further study. However, a careful study opens up new possibilities for therapy with drugs that inhibit the activation of CD44+ cells and/or block complement. For example, using anti-CD44 mAb, it could be shown that collagen-induced arthritis was improved in the rat [28]. Moreover, perhaps a strategy which targets the HA/CD44 interaction could be beneficial.

## 4. Materials and Methods

### 4.1. Clinical Characteristics of the Patients

Patients with a confirmed diagnosis of FSGS (n = 29), MCD (n = 10), MN (n = 10), and IgA nephropathy (n = 11) were recruited for the study.

Most of the diseases debuted with high proteinuria or nephrotic syndrome. Impaired renal function (eGFR CKD-EPI < 60 mL/min/1.73 m^2^) was detected in 17 patients, and normal kidney function was detected in 33 patients (eGFR CKD-EPI > 60 mL/min/1.73 m^2^).

The characteristics of the examined patients are presented in Table 4. The exclusion criteria were active urinary infection, diabetes mellitus, obesity, severe arterial hypertension, liver disease, rheumatic systemic diseases, and stage 5 chronic kidney disease.

### 4.2. Quantitative Analysis of Complement Components in Urine by LC/MRM-MS

The targeted quantitative analysis of complement components in urine was carried out by multiple reaction monitoring (MRM) using liquid chromatography coupled to triple quadrupole mass spectrometer (LC/MRM-MS). In total, 20 synthetic stable isotope-labeled internal standard (SIS) peptides and 20 natural (NAT) synthetic proteotypic peptides were selected for measurements of the 20 corresponding complement components in urine. The SIS peptide mixture was spiked in each urine sample at a balanced concentration which was optimized in the experiments with a dilution series of urine samples with proteinuria. Standard curves were generated using NAT and SIS peptide standards with a pooled urine sample as a matrix as previously described [15].

A total of 10 mL of the middle portion of freshly collected morning urine was centrifuged at 3000 rpm for 15 min immediately after the collection procedure. The supernatant was aliquoted and stored at −20 °C. Urine proteins were precipitated with ice-cold acetone as described previously [15]. In brief, 0.1 mL urine aliquots were quickly thawed, mixed with 0.5 mL of ice-cold acetone, and incubated overnight at −20 °C. The precipitate was centrifuged (20,000× *g*, 10 min) and dissolved in 50 µL of 8 M urea (200 mM Tris-HCL, pH 8.5). Before trypsinolysis, the samples (100 µg of total protein) were reduced with 5 mM of dithiotreitol (30 min, +37 °C) and alkylated in the dark with 20 mM of iodoacetamide (30 min). TPCK-treated trypsin (Worthington, Franklin, OH, USA) was added in an enzyme:protein ratio of 1:25, and hydrolysis was performed at +37 °C overnight. The reaction was quenched by adding formic acid of up to 0.5%. The SIS peptide mixture was spiked in each sample followed by desalting by solid-phase extraction using plates (Oasis HLB 96-well Microelution Plate, Waters, Taunton, MA, USA). The eluate was lyophilized and dissolved in 0.1% formic acid to a concentration of 0.5 mg/mL for further LC-MS/MS analysis. The normalization of the amount of total protein was performed before trypsinolysis and the subsequent MS analysis due to significant variability in the total protein concentrations of the studied urine samples.

All samples were analyzed in duplicate by an HPLC-MS system consisting of an ExionLC™ UHPLC system (ThermoFisher Scientific, Waltham, MA, USA) coupled online to a SCIEX QTRAP 6500+ triple quadrupole mass spectrometer (SCIEX, Toronto, ON, Canada). The loaded sample volume was 10 μL per injection. HPLC separation was carried out using Zorbax Eclipse Plus C18 RRHD column (150 × 2.1 mm, 1.8 μm) (Agilent Technologies, Santa Clara, CA, USA) with gradient elution. Mobile phase A was 0.1% FA in water; mobile phase B was 0.1% FA in acetonitrile. LC separation was performed at a flow rate of 0.4 mL/min using a 53 min gradient from 2 to 45% of mobile phase B. Mass spectrometry measurements were carried out using the MRM acquisition method. The electrospray ionization (ESI) source settings were as follows: ion spray voltage, 4000 V; temperature, 450 °C; ion source gas, 40 L/min.

### 4.3. Histological Study

We used the immunohistochemical peroxidase method to study the expression of CD44 in kidney bioptates. Immunohistochemical staining was performed using BOND-MAX Automated Immunohistochemistry Vision Biosystem (Leica Microsystems GmbH, Wetzlar, Germany) according to the following protocol. Tissue sections were dewaxed with BOND™ Dewax Solution, 100% Alcohol, BOND™ Wash Solution and pre-treated with BOND™ Epitope Retrieval ER2 Solution at 100 °C for 20 min.

After the washing steps, peroxidase blocking was carried out for 5 min using the Detection Kit Peroxide Block (Bond Polymer Refine Detection Kit DS9800 (Leica Microsystems GmbH)). Then, the sections were incubated with primary antibodies for 30 min at room temperature. We used rabbit polyclonal antibody for CD44 (1:200 dilution; BF-9213, Affinity Bioscience, Cincinnati, OH, USA). For the detection of peroxidase activity, DAB-chromogen was used (Bond Polymer Refine Detection Kit DS9800 (Leica Microsystems GmbH)) for 10 min. After the system produced a brown stain, the specimens were washed and immersed in hematoxylin solution for staining nuclei (for 5 min).

CD44 expression in kidney tissue was evaluated, taking into account the number of CD44 positive cells and the intensity of staining, as it was suggested by Remmele and Stegner (Immunoreactive Scale (IRS), shown in Table 5, Figure S1) [29].

### 4.4. Data Analysis

The data were summarized with descriptive statistics. Normality tests were performed with the Shapiro–Wilk test. The results are presented as the number and percentage for categorical variables, as the median and interquartile range (IQR) for continuous variables with a non-normal distribution. The differences between the groups were analyzed by nonparametric methods, the Mann–Whitney and Kruskal–Wallis U-test. We used the χ^2^ and Fisher test to compare the qualitative variables. To determine the correlation relationships between the variables, we used the Spearman correlation (rs). A two-sided *p*-value < 0.05 was considered to indicate statistical significance. Statistical analysis was performed using StatSoft STATISTICA version 10.0 (StatSoft Inc., Tulsa, OK, USA).

For the quantitative analysis of LC/MRM-MS raw data, Skyline Quantitative Analysis software (version 20.2.0.343, University of Washington) was used.

## Figures and Tables

**Table 1:** CD44 expression score in patients with different glomerulopathies.

CD44 Expression Score	FSGS (n = 29)	MCD (n = 10)	MN (n = 10)	IgA Nephropathy (n = 11)	*p* (Two-Tailed Fisher Test)
**Mesangium**	n (%)				
0–1	2 (7)	1 (10)	2 (20)	1 (9)	FSGS/IgAN vs. MCD/MN *p* < 0.05
2–3	7 (24)	7 (70)	5 (50)	1 (9)	
4–8	15 (52)	2 (20)	3 (30)	9 (82)	
9–12	5 (17)	0	0	0	
**PEC**	n (%)				
0–1	9 (31)	7 (70)	8 (80)	3 (27)	FSGS/IgAN vs. MCD/MN *p* < 0.05
2–3	10 (34.5)	3 (30)	2 (20)	6 (55)	
4–8	10 (34.5)	0	0	2 (18)	
9–12	0	0	0	0	
**Podocytes**	n (%)				
0–1	6 (21)	3 (30)	3 (30)	1 (9)	*p* > 0.05
2–3	4 (14)	2 (20)	3 (30)	3 (27)	
4–8	12 (41)	5 (50)	4 (40)	6 (55)	
9–12	7 (24)	0	0	1 (9)	
**Interstitial**	n (%)				
0–1	0	0	0	0	*p* > 0.05
2–3	0	0	0	0	
4–8	9 (31)	4 (40)	3 (30)	1 (9)	
9–12	20 (69)	6 (60)	7 (70)	10 (91)	

Abbreviations: FSGS—focal segmental glomerulosclerosis, MCD—minimal change disease, MN—membranous nephropathy, IgA nephropathy—immunoglobulin A nephropathy, PEC—parietal epithelial cells.

**Table 2:** Correlations between CD44 expression in parietal epithelial cells, mesangial cells ant interstitial cells, and clinical and laboratory data in different glomerulopathies (n = 60).

	CD44 PEC	CD44 MC	CD44 Podocytes	CD44 Interstitial Cells
24-h proteinuria *p*	0.065 0.625	0.092 0.484	0.023 0.362	0.017 0.900
Haematuria *p*	0.030 0.849	0.344 0.022 *	0.291 0.055	0.068 0.660
eGFR CKD-EPI *p*	−0.162 0.217	−0.169 0.192	−0.193 0.136	−0.167 0.199
% of globally sclerotic glomeruli *p*	0.258 0.065	0.273 0.048 *	0.145 0.301	0.233 0.094
Tubulointerstitial fibrosis (TIF) *p*	0.476 0.001 *	0.062 0.660	−0.141 0.313	0.270 0.047 *

∗ *p* < 0.05.

**Table 3:** Correlations (Spearman’s rho and *p*-value) between level of urinary excretion of complement components and degree of glomerulosclerosis. Levels of complement components in urine were determined by LC/MRM mass spectrometry (n = 60).

	C2	C3	C4b	C5	C7	C8alpha	C9	CFB	CFI
Proteinuria g/24 h *p*	0.585 0.088	0.613 0.001 *	0.655 0.001 *	0.600 0.001 *	0.580 0.001 *	0.603 0.001 *	0.551 0.001 *	0.627 0.001 *	0.260 0.203
Creatinine, mkmol/L *p*	0.556 0.015 *	0.459 0.004 *	0.518 0.001 *	0.440 0.019 *	0.524 0.001 *	0.494 0.001 *	0.554 0.001 *	0.464 0.020 *	0.235 0.203
GFR, mL/min/1.73 m^2^ *p*	−0.404 0.088	−0.324 0.050 *	−0.409 0.004 *	−0.288 0.137	−0.430 0.002 *	−0.371 0.009 *	−0.518 0.003 *	−0.363 0.074	−0.297 0.105
Percent of globally sclerotic glomeruli *p*	0.592 0.043 *	0.155 0.439	0.204 0.256	0.332 0.165	0.393 0.022 *	0.238 0.175	0.458 0.037 *	0.391 0.134	0.444 0.034 *
Tubulointerstitial fibrosis, score *p*	0.289 0.389	0.392 0.048 *	0.480 0.006 *	0.432 0.074	0.419 0.017 *	0.607 0.001 *	0.436 0.055	0.346 0.206	0.550 0.008 *

∗ *p* < 0.05.

**Table 4:** Characteristics of the patients.

Parameters	FSGS (n = 29)	MCD (n = 10)	MN (n = 10)	IgA Nephropathy (n = 11)
Age, years	35 (30.0; 55.0)	31.0 (25.3; 39.5)	46.0 (40.5; 51.0)	34.0 (28.0; 41.0)
Gender (male), n (%)	15 (51.7)	1 (10.0)	8 (80)	6 (54.5)
Arterial hypetension, n (%)	21 (72.4)	6 (60.0)	8 (80)	8 (72.7)
Proteinuria, g/24 h	3.92 (2.10; 5.20)	3.05 (1.70; 7.99)	3.9 (2.75; 5.83)	2.54 (2.02; 3.04)
Serum albumin, g/L	31.70 (23.00; 38.40)	30.15 (21.6; 37.82)	28.00 (25.8; 31.00)	35.8 (32.05; 38.75)
Serum protein, g/L	58.4 (47.70; 64.90)	52.35 (43.73; 61.08)	50.75 (45.55; 55.75)	63.3 (58.4; 66.95)
Nephrotic syndrome, n (%)	16 (55.2)	6 (10.0)	8 (72.7)	3 (27.3)
Creatinine, mkmol/L	96.8 (73.37; 162.66)	87.75 (76.08; 101.98)	86.30 (78.05; 97.45)	116 (86.65; 114.00)
eGFR CKD-EPI, mL/min/1.73 m^2^	82.00 (46.00; 101.00)	77.50 (64.00; 89.94)	89.00 (77.5; 97.9)	68.00 (47.0; 76.00)
eGFR < 60 mL/min/1.73 m^2^, n (%)	11 (37.9)	2 (10.0)	1 (10)	5 (45.5)
Steroid-resistant NS, n (%)	11 (37.9)	2 (20.0)	0 (0)	1 (0.09)

Focal segmental glomerulosclerosis, minimal change disease, estimated glomerular filtration rate using the CKD-EPI formula. The table shows the median, in brackets—the 1st and 3rd quartiles.

**Table 5:** Assessment of expression score in points.

Positive Cells (PP)	Intensity of Staining (IS)	IRS (PP × IS)
0	0	0–1 = negative
<10% (=1)	1 (weak)	2–3 = weak
10–50% (=2)	2 (moderate)	4–8 = moderate
51–80% (=3)	3 (strong)	9–12 = strong

## Data Availability

The data supporting the reported results can be found.

## Supplementary Materials

The supporting information can be downloaded at: https://www.mdpi.com/article/10.3390/ijms24087190/s1.
