# Supplementary material for: CD44 Expression in Renal Tissue Is Associated with an Increase in Urinary Levels of Complement Components in Chronic Glomerulopathies
Source: Int J Mol Sci. 2023 Apr 13;24(8):7190. doi: 10.3390/ijms24087190 (PMC10138917; doi:10.3390/ijms24087190)
Supplement: Supplementary file 1 [file ijms-24-07190-s001.zip › ijms-2275357-supplementary figures.pdf]

International Journal of
Molecular Sciences

Supplementary Figure S1. CD44 expression in different compartment of renal tissue.

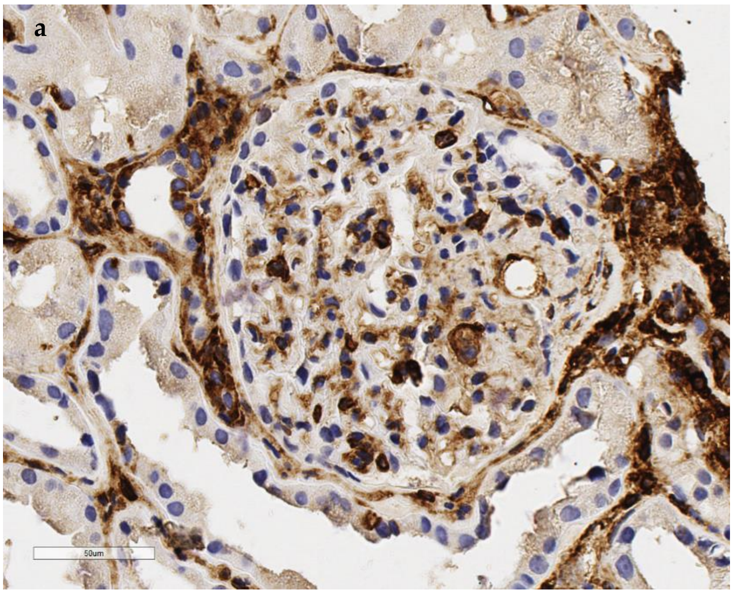	CD44 expression grade 2 in glomerular podocytes, grade 2 in PEC, grade 3 in interstitium, x200.
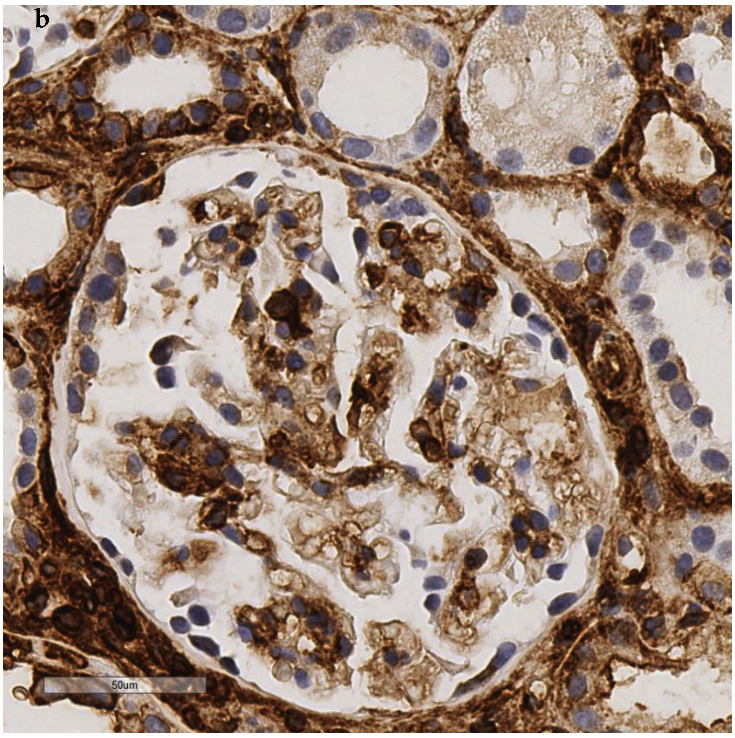	CD44 expression grade 3 in glomerular podocytes and mesangiocytes, grade 1 in PEC, grade 3 in interstitium, x200.

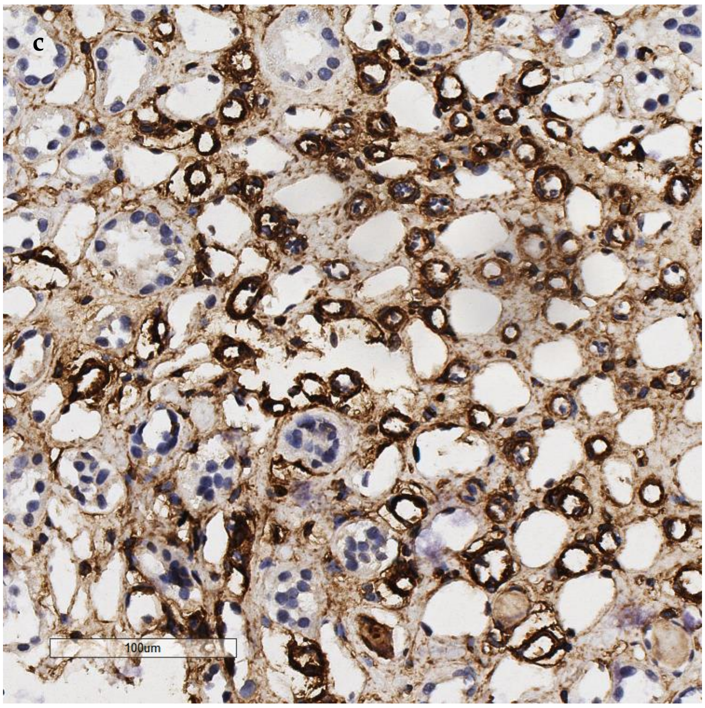	CD44 expression grade 3 in the tubular basement membrane of the straight tubules, *x*200.
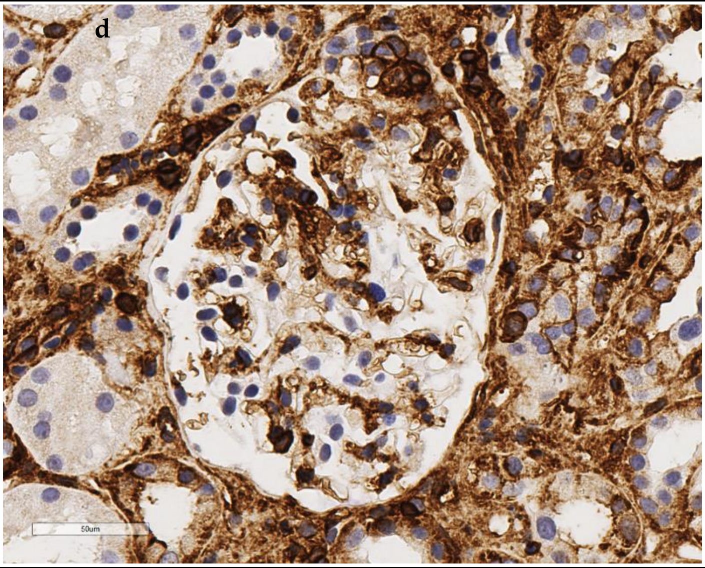	CD44 expression grade 3 in glomerular podocytes and mesangiocytes, grade 2 in PEC, grade 3 in interstitium, x200.

Supplementary Figure S2. Correlations between different components complement levels, glomerulosclerosis and tubulointerstitial fibrosis

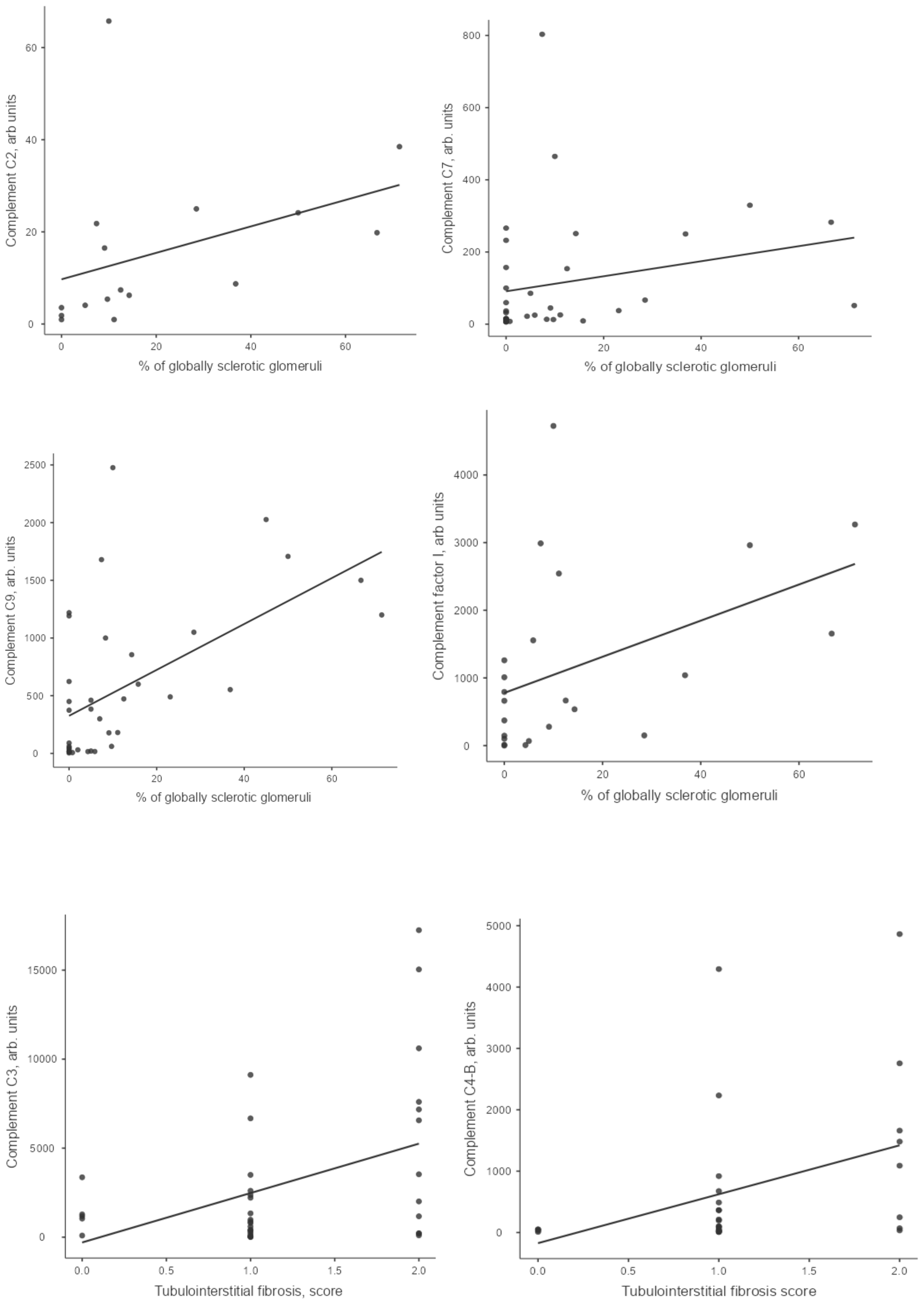

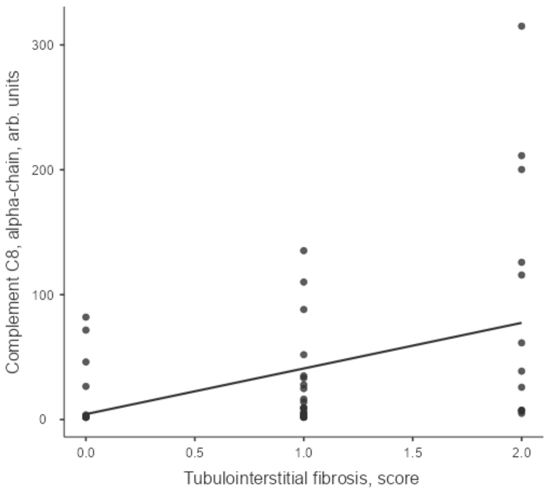
Complement C8, alpha-chain, arb. units
300
200
100
0
0.0
0.5
1.0
1.5
2.0
Tubulointerstitial fibrosis, score

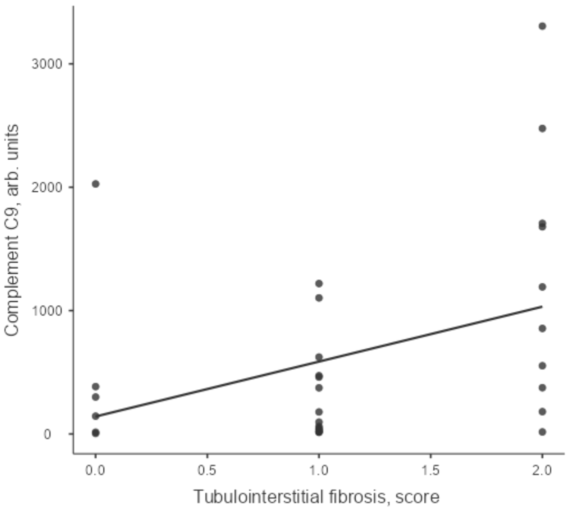
Complement C9, arb. units
3000
2000
1000
0
0.0
0.5
1.0
1.5
2.0
Tubulointerstitial fibrosis, score

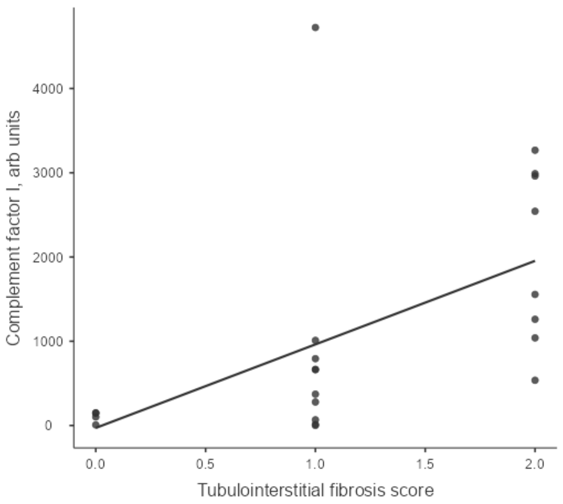
Complement factor I, arb units
4000
3000
2000
1000
0
0.0
0.5
1.0
1.5
2.0
Tubulointerstitial fibrosis score
